# Acceptability of Telemedicine Among Parents of Adolescent Patients in an Adolescent Clinic: Cross-sectional Survey Study

**DOI:** 10.2196/39704

**Published:** 2022-12-21

**Authors:** Adetola Olateju, Marbella Cervantes, Nadia Dowshen, Lisa M Kuhns, Cherie Priya Dhar

**Affiliations:** 1 Northwestern University Feinberg School of Medicine Chicago, IL United States; 2 Ann and Robert H Lurie Children's Hospital of Chicago Chicago, IL United States; 3 Department of Pediatrics Perelman School of Medicine University of Pennsylvania Philadelphia, PA United States; 4 Craig-Dalsimer Division of Adolescent Medicine Children's Hospital of Philadelphia Philadelphia, PA United States

**Keywords:** adolescent medicine, telemedicine, acceptability, privacy, confidentiality, satisfaction, caregivers

## Abstract

**Background:**

Since the beginning of the COVID-19 pandemic, new literature has described the perceptions of adolescent patients on the use of telemedicine for their health care, but less attention has been devoted to parents’ and caregivers’ perspectives on telemedicine usage for their adolescents. Parents’ perspectives are important, as they undoubtedly influence how children learn to make decisions about their health care.

**Objective:**

This study describes the level of acceptability (measured based on accessibility and satisfaction) expressed by caregivers of adolescent patients with regard to telemedicine visits in an urban adolescent medicine practice.

**Methods:**

A cross-sectional survey was sent electronically to parents and guardians of patients aged <18 years who completed outpatient telemedicine visits to an adolescent medicine practice in Chicago, Illinois, from March 2020 to February 2021. The questions focused on accessibility and satisfaction. The data were analyzed to describe response frequencies.

**Results:**

Among a sample of 71 survey respondents, the vast majority reported that telemedicine was very easy to use (58/71, 82%) and was at least as convenient as in-person visits (70/71, 99%). Over 90% of respondents reported that their adolescents’ needs were addressed (69/69, 100%) and that they were at least as comfortable with the level of privacy and the confidential conversations between their adolescents and medical providers in telemedicine visits (65/71, 92%) as they were with those in in-person visits.

**Conclusions:**

Our findings suggest that parents and guardians find telemedicine to be an acceptable way for their children and adolescents to receive appropriate health care.

## Introduction

When the SARS-CoV-2 pandemic began in the United States on March 2020, telemedicine services grew exponentially to meet patient care needs. Since then, studies on the utilization of telemedicine by adult and pediatric populations have suggested that telemedicine is an acceptable alternative to in-person visits and that patients are mostly satisfied with the use of telemedicine, largely due to increased convenience [1,2]. Adolescent medicine is a distinct area of pediatrics that involves specialized care with the added complexity of confidentiality and privacy, as it relates to the receipt of care by minors [3]. Studies to date suggest that adolescents are satisfied with the convenience and accessibility of telemedicine visits [4-6] and have few concerns about privacy and confidentiality [7]. Evidence indicates that most adolescents can find a quiet room in which to conduct a telemedicine visit, with few describing a lack of privacy or the fear of being overheard by someone else [6]. These studies however are mainly focused on youth perspectives; less attention is paid to the perceptions of parents and caregivers [4,8,9]. Caregivers are important stakeholders in the care of adolescents, as they make and share health decisions with their adolescents while also allowing confidential health care conversations between their adolescents and medical providers [10,11]. A study published in 2021 delineated adolescent and caregiver perspectives for care in disordered eating and reproductive health care. In that study, adolescents were able to locate a private space to conduct telehealth visits, and caregivers found telehealth to be noninferior to in-person visits with regard to privacy, communication, the discussion of test results, and mood-related issues [12]. Our study aims to add to current literature by describing caregiver perceptions on the acceptability of adolescent telemedicine visits in terms of both perceived accessibility and satisfaction. We hypothesized that caregivers would find telemedicine to be an acceptable means of health care delivery for their adolescents.

## Methods

### Study Design

In this study, we collected cross-sectional data via a self-administered survey that was sent electronically to caregivers of patients aged <18 years who completed outpatient telemedicine visits via StarLeaf (StarLeaf Ltd)—a teleconferencing application—with the Division of Adolescent Medicine at Lurie Children’s Hospital of Chicago between March 2020 and February 2021. Some portions of the visits may have been conducted without the guardians being present (ie, the physician was alone with the adolescent), although the times when this occurred during the visits varied by medical provider. During the data collection period, survey invitations were sent via email to a convenient sample of parents and guardians (ie, those who completed telemedicine visits and had an email address on file) within 72 hours of each visit. Survey invitations were emailed once and remained active until the end of the data collection period. Surveys were completed via REDCap (Research Electronic Data Capture; Vanderbilt University) and included demographic characteristics (eg, race), the reason for the health care visit, a history of the receipt of telemedicine visits (ie, visits for the parent’s or guardian’s own health care or visits for their adolescent’s health care), and questions for assessing the accessibility of and satisfaction with the telemedicine visit (components of acceptability). These questions used a 5-point scale for comparing the accessibility of and satisfaction with telemedicine visits versus in-person visits. The data were summarized by using frequencies of responses and were further dichotomized into the following two groups: respondents who either preferred telemedicine over in-person visits or had no preferences for the type of visit and respondents who preferred in-person visits over telemedicine.

### Ethics Approval

The study protocol was reviewed and approved by Lurie Children’s institutional review board (reference number: 2020-3737) with a waiver of documentation of consent.

## Results

**Figure 1:**
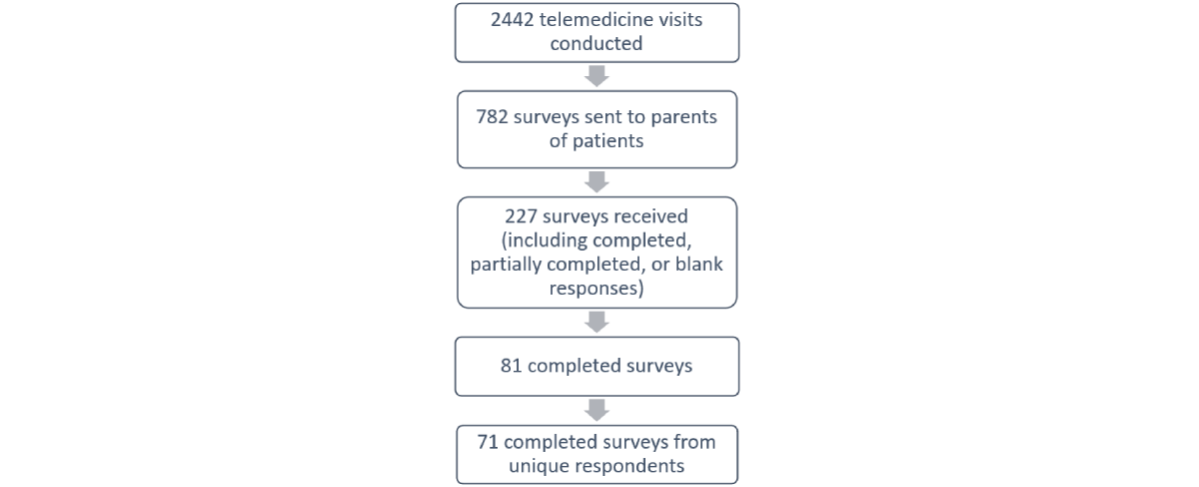
Flow of adolescent medicine surveys sent and received.

A total of 2442 telemedicine visits occurred between March 2020 and February 2021. A convenient sample of 782 surveys were sent to parents and guardians, and a total of 227 surveys were received—a response rate of 29% (227/782). Of the 81 surveys that were returned completed, 71 were from unique participants (Figure 1). For those who completed more than 1 survey due to multiple encounters, the responses from the first telemedicine encounter were used in the analysis. REDCap software was used for the collection of survey data. SPSS Statistics 27 (IBM Corp) was used for the analysis of data. The demographic data of respondents are provided in Table 1. The majority (61/71, 86%) self-identified as White, while the rest of the respondents self-identified as Black or African American, Native American or Alaskan Native, Native Hawaiian or Other Pacific Islander, Asian, or other. The average age of caregivers was 47 years. Further, 72% (51/71) of parents had used telemedicine previously. Visits were for primary care; sexual, menstrual, or reproductive health; mental health; or gender transition–related care. The most common reason for telemedicine visits was the receipt of gender-affirming care (46/71, 65%). Patients who received gender-affirming care were part of the Gender Development Clinic; they may have been starting pubertal blockade therapy or gender-affirming hormones, or they started these processes and were seeking follow-up care.

With regard to accessibility and satisfaction, survey items and their response frequencies are described in Table 2. All caregivers reported that video visits were somewhat easy to use or very easy to use. The majority (60/71, 85%) reported that if video visits had not been available, they would have waited until in-person appointments were available or until the COVID-19 pandemic ended to receive care (ie, rather than seek immediate care elsewhere or use the emergency department).

Almost all respondents (69/71, 97%) reported that telemedicine was at least equally as convenient as an in-person visit. Further, the vast majority (70/71, 99%) indicated that their adolescents’ concerns were addressed at least as well as they would have been in in-person visits, reported that they were at least as comfortable with leaving the room (69/71, 97%; ie, to allow a confidential conversation between the physician and adolescent) as they would have been for an in-person visit, and felt at least as comfortable with the level of privacy (65/71, 92%) as they were with that of an in-person visit. A total of 89% (63/71) of the respondents reported that they would be very likely or extremely likely to recommend telemedicine to others even after the COVID-19 pandemic was over.

**Table 1:** Demographic characteristics of caregivers of adolescents (n=71).

Characteristics				Respondents
**Race^a^, n (%)**				
	White		61 (86)	
	Black or African American		6 (9)	
	Native American or Alaskan Native		0 (0)	
	Native Hawaiian or Other Pacific Islander		0 (0)	
	Asian		3 (4)	
	Other		2 (3)	
**Hispanic or Latinx, n (%)**				
	Yes		8 (11)	
	No		63 (89)	
Age (years), mean (range)				47 (34-66)
**Reason for visit^a^, n (%)**				
	Sexual, menstrual, or reproductive health		13 (18)	
	Gender-related care		46 (65)	
	Mental health		17 (24)	
	Primary care		5 (7)	
	Substance use prevention program		0 (0)	
**“During the video visits, was your child referred for additional services? (Bloodwork, labs, a physical exam, pharmacy BP, etc),” n (%)**				
	**Yes**		27 (38)	
		Sexual, menstrual, or reproductive health	2 (7)	
		Gender-related care	23 (77)	
		Mental health	2 (7)	
		Primary care	3 (10)	
		Substance use prevention program	0 (0)	
	No		44 (62)	

^a^Respondents checked all response options that applied.

**Table 2:** Caregiver acceptability of telemedicine for adolescent health visits (n=71).

Survey questions and responses				Respondents, n (%)
**Accessibility**				
	**“How easy or difficult was it to use the video visit system?”**			
		“Very difficult”	0 (0)	
		“Somewhat difficult”	0 (0)	
		“Somewhat easy”	11 (15)	
		“Very easy”	58 (82)	
		No response	2 (3)	
	**“If a video visit for your child was not available today, what would you have done?”**			
		“Waited to make an adolescent medicine in-person appointment”	52 (73)	
		“Gone to the emergency department”	0 (0)	
		“Looked for a provider outside the Lurie system”	1 (1)	
		“Waited to seek care after COVID-19”	8 (11)	
		“I do not know”	10 (14)	
**Satisfaction**				
	**“The visit was convenient for me.”**			
		“Telehealth much better than in-person”	42 (59)	
		“Telehealth somewhat better than in-person”	12 (17)	
		“Telehealth about the same as in-person”	16 (23)	
		“Telehealth somewhat worse than in-person”	1 (1)	
		“Telehealth much worse than in person”	0 (0)	
	**“I felt my child’s concerns were addressed.”**			
		“Telehealth much better than in-person”	4 (6)	
		“Telehealth somewhat better than in-person”	1 (1)	
		“Telehealth about the same as in person”	65 (92)	
		“Telehealth somewhat worse than in-person”	1 (1)	
		“Telehealth much worse than in-person”	0 (0)	
	**“I felt comfortable leaving the room.”**			
		“Telehealth much better than in-person”	8 (11)	
		“Telehealth somewhat better than in-person”	2 (3)	
		“Telehealth about the same as in person”	59 (83)	
		“Telehealth somewhat worse than in-person”	0 (0)	
		“Telehealth much worse than in-person”	2 (3)	
	**“I felt comfortable with the privacy of the video visit.”**			
		“Telehealth much better than in-person”	8 (11)	
		“Telehealth somewhat better than in-person”	3 (4)	
		“Telehealth about the same as in person”	54 (76)	
		“Telehealth somewhat worse than in-person”	4 (6)	
		“Telehealth much worse than in-person”	2 (3)	
	**“How likely are you to recommend video visits to a family member or friend after the COVID-19 crisis?”**			
		“Not at all likely”	2 (3)	
		“Somewhat likely”	6 (8)	
		“Very likely”	24 (34)	
		“Extremely likely”	39 (55)	

## Discussion

### Principal Findings

In this project, we aimed to describe the level of acceptability (measured based on accessibility and satisfaction) expressed by caregivers of adolescent patients with regard to telemedicine use during the first year of the COVID-19 pandemic. Our results show a high level of acceptability for telemedicine among caregivers of adolescent patients receiving predominantly gender-related specialty care. Prior to the COVID-19 pandemic, during an equivalent time period at the Division of Adolescent Medicine at Lurie Children’s Hospital, 53.3% (1301/2442) of telemedicine encounters were conducted for gender-related care. Similarly, in this study, gender-related care represented 65% (46/71) of care visits, suggesting that telemedicine is crucial for accessing gender-related care after the pandemic. Prior studies of gender-diverse youth have shown that youth have an interest in receiving care through telehealth [6], and studies of young adult patients and caregivers of youth accessing gender transition–related services have shown preliminary data indicating that telehealth visits are more acceptable and convenient than in-person visits [13]. For all adolescent health–related visits, our results similarly indicate that for many caregivers of adolescent patients, telemedicine is at least equally as acceptable and satisfactory as in-person medical visits. Of the 71 respondents, only 1 felt as if their adolescent’s needs were not addressed by their medical provider during the telemedicine visit. There were minimal issues with the use of technology, and all respondents found the video system easy to use.

Confidentiality is a cornerstone of adolescent health issues; the American Academy of Pediatrics has provided guidance on confidentiality for adolescent telehealth visits [14]. All caregivers and families should be reminded that confidential time alone without a parent should be expected for every telemedicine encounter, just as it would for in-person visits. Physicians should discuss the restrictions of confidential care (eg, if the adolescent’s safety or someone else’s safety is in danger) and ensure that confidential information is housed in the electronic health record appropriately to prevent a parent from accessing information that is not intended to be shared with them [14]. For sexual and gender minority youth in particular, the issue of privacy is critical for improved health care outcomes. Studies related to privacy and telehealth for adolescents have shown mixed results. A survey study conducted at a pediatric center that provides gender-affirming care found no differences in caregiver perceptions of privacy between in-person visits and video visits [13]. However, an adolescent medicine clinic that cares for patients with disordered eating and reproductive health concerns found that 22% of youths believed that the privacy of telehealth visits was inferior to that of in-person visits, whereas only 2.5% of caregivers shared that belief [12]. In our study, the majority of parents (65/71, 92%) did not have concerns about privacy during telehealth visits. Although our study did not elicit adolescents’ experiences or their perceptions of privacy, parents’ comfort with leaving the room and allowing for private discussions between adolescents and medical providers, as well as the overall privacy of visits, is a critical component of acceptability and high-quality patient care.

Despite the high acceptability of and satisfaction with telemedicine among the group surveyed, there may be some disadvantages. Telemedicine does not allow for thorough physical examinations, the recording of vitals, or blood work, which can be critical parts of adolescent care. Barney et al [15] noted that some reproductive health services cannot be conducted via telemedicine, including sexually transmitted infection screening, the insertion of contraceptive devices, and gynecologic examinations. Of the 13 caregivers in our study who had adolescents with reproductive health concerns, only 2 stated that they were referred for additional services. In many cases, medical providers were able to provide adequate care via the video system that did not require an in-person physical examination. Further, telemedicine can be limited by access to a device or an internet connection, which is necessary for participating in a telemedicine visit. Although many of our respondents did not experience any technical difficulties during the video visits, it is unclear if this would be true for the general population seeking adolescent care or if this was influenced by a lack of demographic diversity within the group of respondents.

### Limitations

This study is limited in terms of the small sample size and low responsiveness to the survey invitation. However, the sample size of this study is comparable to those of other studies evaluating telemedicine in adolescent populations. The individuals who completed the survey might not be representative of the larger group of families seeking a variety of adolescent health services via telemedicine, as most respondents self-identified as White (61/71, 86%) and had adolescents who were seeking gender-related health care (46/71, 65%).

### Conclusions

In response to the COVID-19 pandemic, telemedicine has undoubtedly been implemented more than ever before. Among parents of adolescents receiving specialty care at our institution, telemedicine is viewed as an acceptable way to receive medical care with minimal concerns for privacy and confidentiality. Further studies are needed to assess telemedicine’s acceptability in more diverse populations and its appropriateness for addressing the variety of adolescent health needs.

### Implications and Contributions

This paper contributes to the growing body of literature about the acceptability of telehealth by highlighting parent perspectives on the receipt of adolescent-specific telehealth services for their adolescents.

## Abbreviations

REDCap: Research Electronic Data Capture
